# Estimation of Aortic Valve Calcium Score Based on Angiographic Phase Versus Reduction of Ionizing Radiation Dose in Computed Tomography

**DOI:** 10.3390/life11070604

**Published:** 2021-06-23

**Authors:** Paweł Gać, Bartłomiej Kędzierski, Piotr Macek, Krystyna Pawlas, Rafał Poręba

**Affiliations:** 1 Department of Hygiene, Wroclaw Medical University, Mikulicza-Radeckiego 7, PL 50-368 Wroclaw, Poland; krystyna.pawlas@umed.wroc.pl; 2 Centre for Diagnostic Imaging, 4th Military Hospital, Weigla 5, PL 50-981 Wroclaw, Poland; 3 Center for Diagnostic Imaging, University Clinical Hospital in Wrocław, Borowska 213, PL 50-556 Wroclaw, Poland; bkedzierski@usk.wroc.pl; 4 Department of Internal Medicine, Occupational Diseases, Hypertension and Clinical Oncology, Wroclaw Medical University, Borowska 213, PL 50-556 Wroclaw, Poland; piotr.macek@student.umed.wroc.pl (P.M.); rafal.poreba@umed.wroc.pl (R.P.)

**Keywords:** aortic valve calcium score, TAVI, radiation dose

## Abstract

The aim of the study was to evaluate the estimation efficacy of aortic valve calcium score (AVCS) based on the multislice computed tomography (MSCT) angiographic phase. The evaluation of the reduced amount of ionizing radiation dose was performed because of this estimation. The study included 51 consecutive patients who qualified for transcatheter aortic valve implantation (TAVI) (78.59 ± 5.72 years). All subjects underwent MSCT: in the native phase dedicated to AVCS as well as angiographic phases aimed to morphologically assess the aortic ostium and arterial accesses for TAVI. Based on the native phase, an AVCS assessment was performed for axial reconstructions at 3.0 mm and 2.0 mm slice thickness (AVCS_native3.0_ and AVCS_native2.0_). Based on the angiographic phase AVCS was estimated for axial reconstruction at 0.6 mm slice thickness with increased values of lesion density in aortic valve cusps/aortic valve annulus, which is considered a calcification, from a typical value of 130 HU to 500 HU and 600 HU (AVCS_CTA0.6 500 HU_ and AVCS_CTA0.6 600 HU_). Mathematical formulations were developed, allowing for AVCS native calculation based on AVCS values estimated based on the angiographic phase: AVCS_native3.0_ = 813.920 + 1.510 AVCS_CTA0.6 500 HU_; AVCS_native3.0_ = 1235.863 + 1.817 AVCS_CTA0.6 600 HU_; AVCS_native2.0_ = 797.471 + 1.393 AVCS_CTA0.6 500 HU_; AVCS_native2.0_ = 1228.310 + 1.650 AVCS_CTA0.6 600 HU_. The amount of a potential reduction in dose length product (DLP) in the case of AVCS estimation was 4.45 ± 1.54%. In summary, relying solely on the angiographic phase of MSCT examination before TAVI, it is possible to conclusively estimate AVCS. This estimation results in a marked reduction in radiation dose in MSCT.

## 1. Introduction

Aortic valve stenosis is the most common primary defect of heart valves [1]. The incidence of aortic stenosis increases with age and in people between 50 and 59 years of age it affects 0.2% of the population; in persons older than 75, as many as 2.8% of the population; in people older than 80 as many as 9.8% of the population [2]. Symptomatic aortic valve stenosis is associated with 50% mortality rate in the period of 2 years [3].

The pathomechanism of aortic valve stenosis development basically involves the damage of the endothelium-covering valve cusps, the migration of lipoproteins to endothelial space, the development of inflammatory reaction, resulting in aortic valve calcification [4,5]. The calcification of the aortic valve is the underlying pathomechanism of aortic stenosis development. Single calcification foci are regarded as the mild calcification of aortic valve, multiple calcification foci indicate moderate calcification and the complete calcification of cusps, and their thickening is referred to as the severe calcification of the aortic valve [6].

Aortic valve stenosis is a progressive disease process. Generally, the treatment of severe aortic valve stenosis involves a cardiac surgical procedure, i.e., the replacement of aortic valve [7]. An alternative treatment, particularly in elderly patients and/or patients with comorbidities, is transcatheter aortic valve implantation (TAVI) [8,9].

The basic diagnostic method in the qualification procedure for TAVI is the multislice computed tomography (MSCT) of the heart and large vessels [10]. It allows for the evaluation of sizes and the geometry of aortic valve, anatomy of aortic ostium as well as the evaluation of vascular accesses for TAVI. The MSCT also allows for the evaluation of aortic valve calcium score (AVCS) [11]. AVCS is a mathematically estimated, quantitative, non-unitary parameter which characterizes the total amount of calcification within the aortic valve region [12]. Based on the guidelines of scientific associations, AVCS is used to differentiate the degrees of aortic stenosis with an aortic ostium surface area below 1.0 cm^2^, a low gradient (<40 mmHg) and maintained left ventricular ejection fraction [13].

The basic reservation regarding MSCT examination for TAVI qualification is the inherent necessity to use ionizing radiation. In the recent years, the doses of radiation have been significantly reduced in MSCT examinations; however, in accordance with the ALARA (As Low as Reasonably Achievable) rule, the methods aimed at a further reduction in the ionizing radiation dose during the procedures utilizing ionizing radiation should be always sought for, while optimizing the adequate quality of the obtained diagnostic images and sometimes sacrificing negligible quality reduction in such images [14,15].

The AVCS assessment, useful during qualification for TAVI in the above-mentioned clinical situations, requires an additional native phase of the MSCT study [11]. The hypothesis of technically feasible, reliable estimation of AVCS value based on the angiographic phase of MSCT examination with the simultaneous omission of the native phase of the examination appears to be of certain importance. In case of a positive verification of the assumed hypothesis it seems reasonable to evaluate the amount of the potential reduction in radiation dose related to application of this estimation.

The aim of the study was to evaluate the estimation efficacy of AVCS based on the angiographic phase of the MSCT examination of the heart and large vessels.

Moreover, the potential reduction in the ionizing radiation dose was evaluated due to the estimation of the value of AVCS based on the angiographic phase instead of performing the native phase of the MSCT.

## 2. Materials and Methods

### 2.1. Study Group

The study group included 51 consecutive patients who underwent MSCT examination of the heart and large vessels with AVCS assessment at CT Lab to qualify for TAVI in 2019.

Group size was determined using a sample size calculator. The selection conditions were as follows: population size 38 million, fraction size 0.1, maximum error 10%, confidence level 95%. The required minimum size of the study group was 35. During the analyzed period, 51 patients at the CT Lab were examined, hence the final size of the study group.

### 2.2. Study Methodology

The current study was performed as part of the project entitled: “Possibility to optimize ionizing radiation procedure in MSCT examination in the qualification procedure for TAVI.”

Medical history was obtained from all the included patients, their basic anthropometric parameters were measured and assessment of the MSCT of the heart and large vessels with AVCS was performed.

#### 2.2.1. Basic Anthropometric Measurements

The study group underwent basic anthropometric measurements: their age, gender and body weight were defined. Based on the formula: body weight (kg)/height (m)^2^, body mass index (BMI) was calculated. Since the measured anthropometric parameters, subgroups of men and women were selected and included the patients with normal body weight, overweight and obesity, as well as elderly and senile patients. Typical criteria of overweight and obesity, based on BMI, were assumed: BMI between 20 and 24.99 kg/m^2^ as normal body mass, BMI between 25 and 29.99 kg/m^2^ was classed as overweight and a BMI over 30 kg/m^2^ as obesity. In accordance with the guidelines of the World Health Organization, elderly patients were considered persons aged 60–74 and senile patients in the age range 75–90 [16].

#### 2.2.2. MSCT of the Heart and Large Vessels

In all study patients, the MSCT of the heart and large vessels with AVCS assessment was performed. MSCT examination was performed on a 128-slice CT (Somatom Definition AS+, Siemens Healthcare, Erlangen, Germany) following the standard protocol. According to the protocol, the following procedures were performed: topogram, native phase dedicated to the evaluation of AVCS, pre-monitoring and monitoring at trachea bifurcation with image acquisition triggered by post-contrast saturation ROI placed at ascending aorta at 100 HU, ECG-gated angiographic phase dedicated to the morphological evaluation of aortic ostium and angiographic phase dedicated to the imaging of potential arterial accesses for TAVI. Native phase and angiographic phase acquisitions dedicated to the morphological evaluation of aortic ostium also involved tracheal bifurcation at the level of costophrenic angles, and angiographic phase dedicated to imaging of potential arterial accesses for TAVI procedure involved the area ranging from shoulders to the lesser trochanter of the femur. Exposure at 120 kV X-ray lamp was used and mAs values were variable. The volume of the contrast agent administered intravenously was adjusted depending on the examination stage. Using an automated syringe, 70–100 mL of iodinated, non-ionic contrast agent (joheksol, 350 mg iodine/mL; Omnipaque 350, GE Healthcare Oslo, Norway) was administered into the cubital fossa vein at the injection speed of 4.0 mL/s. In the native phase of the examination axial section, reconstructions of 3.0 mm and 2.0 mm slice thicknesses were performed; in the angiographic phase dedicated to morphological evaluation of aortic ostium axial section reconstructions of 3.0 mm and 0.6 mm slice thicknesses were performed; in the angiographic phase dedicated to the imaging of potential arterial access for TAVI procedure, axial section reconstructions of 3.0 mm and 1.0 mm slice thicknesses were obtained. Moreover, angiographic phases of MSCT examination included secondary multiplanar reconstructions (MPR) in frontal and sagittal sections, as well as maximum intensity projection (MIP) and volume rendering technique (VRT).

#### 2.2.3. Evaluation of Aortic Valve Calcium Score

The evaluation of AVCS was performed retrospectively using the syngo.CT CaScoring application (Siemens Healthcare, Erlangen, Germany).

**Figure 1:**
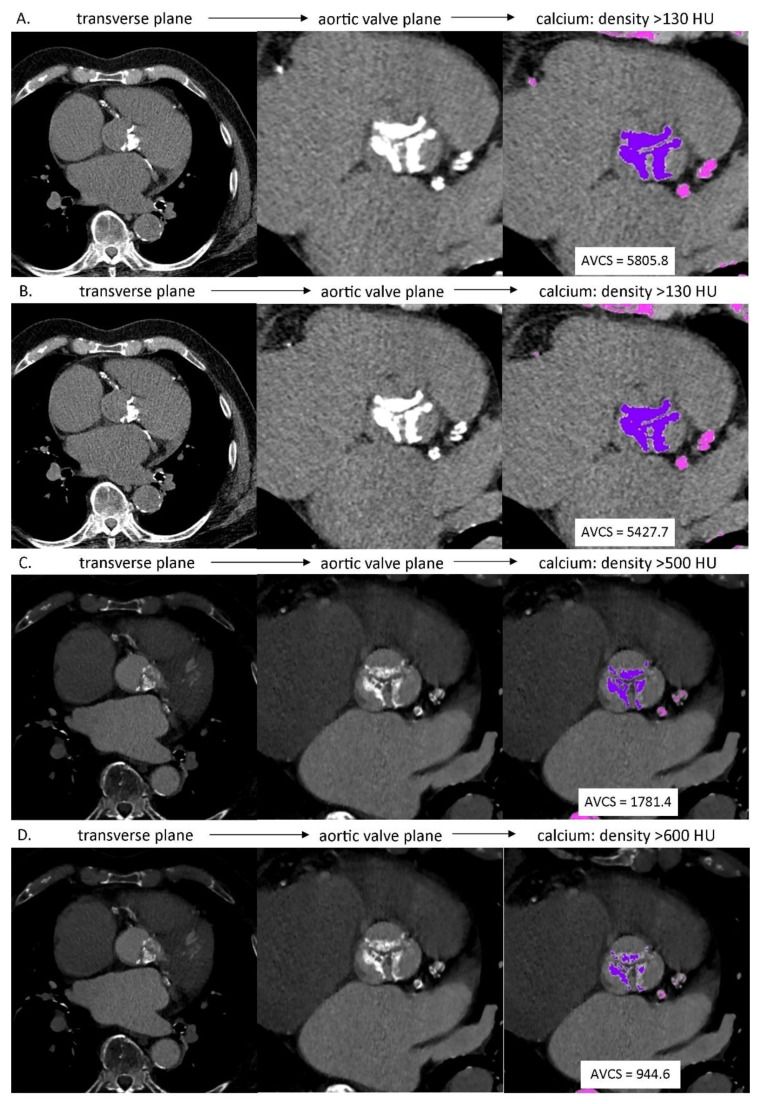
AVCS assessment in MSCT: (**A**) native phase, axial reconstruction, slice thickness: 3.0 mm, calcium detection threshold: density >130 HU; (**B**) native phase, axial reconstruction, slice thickness: 2.0 mm, calcium detection threshold: density >130 HU; (**C**) angiographic phase, axial reconstruction, slice thickness: 0.6 mm, calcium detection threshold: density >500 HU; (**D**) angiographic phase, axial reconstruction, slice thickness: 0.6 mm, calcium detection threshold: density >600 HU.

Based on the native phase of MSCT examination, the semi-automatic evaluation of AVCS was performed for axial reconstructions of 3.0 mm and 2.0 mm slice thicknesses (AVCS_native3.0_ and AVCS_native2.0_, respectively). The lesions were considered calcified, in accordance with Agatston algorithm, if their density exceeded 130 HU. The lesions suggested by the application as calcified were categorized as the lesions in aortic valve cusps/aortic valve annulus or as the lesions outside these structures. The aortic valve calcification categorization for each patient was verified by the consensus of the same two evaluating radiologists. Based on the above categorization the application performed calculations of AVCS values (Figure 1A,B).

Based on the angiographic, ECG-gated, best diastolic phase of MSCT examination dedicated to the morphological evaluation of the aortic valve, AVCS was estimated for axial reconstructions of 0.6 mm slice thickness, with increased values of lesion density in aortic valve cusps/aortic valve annulus considered to be calcified from a typical value of 130 HU to 500 HU and 600 HU (AVCS_CTA0.6 500 HU_ and AVCS_CTA0.6 600 HU_, respectively). The 500 HU and 600 HU values were subjectively selected to differentiate the calcified lesions in aortic valve cusps/aortic valve annulus from the contrasted lumen of left ventricular outflow tract and/or aortic root. Apart from that, calculations of estimated value of AVCS were performed analogically to the above-described method regarding determination of native AVCS value (Figure 1C,D).

Assessment of AVCS in the native phase and assessment of AVCS in the angiographic phase were separated in time. At the time of the AVCS assessment in the angiographic phase, the investigators had no information about the value of the AVCS in the native phase.

#### 2.2.4. Severity Criteria of Aortic Valve Stenosis

AVCS_native3.0_ values were used to estimate the probability of severe aortic valve stenosis. AVCS_native3.0_ values over 3000 in men and 1600 in women were assumed as indicators of a highly probable severe aortic stenosis. AVCS_native3.0_ values over 2000 in men and 1200 in women were considered as indicators of a probable severe aortic stenosis. The patients whose AVCS_native3.0_ values were lower than 1600 in men and 800 in women were classified as improbable to have a severe aortic stenosis [13].

#### 2.2.5. Ionizing Radiation Dose

Ionizing radiation dose in the analyzed MSCT examinations was characterized by the automated reading of the measurements performed during image acquisition by CT device. The radiation dose was formulated by computed tomography dose index (CTDIvol) and dose length product (DLP) for the native phase dedicated to AVCS evaluation and the angiographic phase used to morphologically evaluate the aortic ostium. Moreover, the total DLP dose was determined for MSCT examination of the hearth and large vessels with the AVCS assessment.

#### 2.2.6. Statistical Analysis

Statistical analysis was performed using “Dell Statistica 13” tool (Dell Inc., Round Rock, TX, USA). Mean arithmetic values (X), medians (Me), minimum (Min) and maximum (Max) values as well as standard deviations (SD) were calculated for quantitative variables of the designated parameters. The results for qualitative (nominal) variables were expressed as absolute figures (*n*) and percentage configurations (%). Distribution of variables was checked using the Shapiro–Wilk test. To determine relationships between the studied variables correlation analysis as well as regression analyses were performed. In the case of quantitative variables of normal distribution, the Pearson correlation coefficient was used. The mathematical formulas for computing the native AVCS from the known AVCS values measured during the angiographic phase of the MSCT were determined by univariate and multivariate regression analysis. The parameters of the obtained formulas in regression analysis were estimated using the least square method. Evaluations of predictive accuracy of the tests were performed using ROC analysis. In the comparative analysis of the selected subgroups, in the case of independent quantitative variables of normal distribution for further statistical analysis, the *t*-test for independent variables or analysis of variance—ANOVA (parametric univariate)—was used. The results at the level of *p* < 0.05 were considered statistically significant.

## 3. Results

### 3.1. Study Group Characteristics

The women constituted 49.02% and the men constituted 50.98% of the study population. The mean age of the included patients was 78.59 ± 5.72 years of age. The basic anthropometric parameters of the study group are presented in Table 1.

### 3.2. Aortic Valve Evaluation in a MSCT before TAVI

96.08% of patients were found to have a tricuspid aortic valve, whereas 3.92% of patients had a bicuspid aortic valve. Average sizes of aortic valve annulus and aortic root were 26.64 ± 3.21 mm and 32.16 ± 4.28 mm, respectively, and the height of the aortic root was 19.88 ± 3.79 mm. Standard parameters of aortic valve evaluation in MSCT examination performed before TAVI in the study group are presented in Table 2.

### 3.3. AVCS in a MSCT before TAVI

AVCS, depending on the thickness of native image reconstructions, in the study group of patients was 3690.54 ± 2378.82 in the case of 3.0 mm (AVCS_native3.0_) slice thickness and 3457.03 ± 2190.81 in the case of 2.0 mm (AVCS_native2.0_) slice thickness. Based on AVCSnative3.0 the estimated probability of severe aortic stenosis in 58.82% of the study group was highly probable, in 88.23% it was probable, and in 3.92% it was improbable. The mean value of the blood pool density in the lumen of the left ventricle and the aortic bulb was 380.84 ± 113.33 HU and 392.21 ± 129.12 HU. AVCS estimated based on CT angiographic phase, depending on the increased threshold of calcification detection, was 2068.62 ± 1422.23 with the calcification detection threshold increased up to 500 HU (AVCS_CTA0.6 500 HU_) and 1372.39 ± 1044.53 with the calcification detection threshold increased to 600 HU (AVCS_CTA0.6 600 HU_). With both the abovementioned calcification detection thresholds, AVCS value estimation was possible in 76.47% and 98.04% of examinations, respectively. In the remaining cases, the indicated density thresholds were insufficient to differentiate between calcium and contrasted blood pool; consequently, syngo.CT CaScoring failed to generate AVCS results. AVCS values in MSCT examinations before TAVI in the tested group of patients are presented in Table 3.

### 3.4. Ionizing Radiation Dose in a MSCT before TAVI

Table 3 also shows the parameters characterizing the ionizing radiation dose in the analyzed MSCT examinations. The mean values of CTDIvol and DLP of native phase dedicated to AVCS evaluation were 2.35 ± 2.55 mGy and 30.50 ± 26.85 mGycm, respectively. Analogous parameters for the angiographic phase, dedicated to morphological evaluation of aortic ostium were 29.58 ± 45.63 mGy and 324.18 ± 328.41 mGycm. Regarding the phase of the examination dedicated to the evaluation of vascular access in the TAVI procedure, the total radiation dose in the analyzed MSCT examinations was, on average, 697.94 ± 472.17 mGycm.

### 3.5. Correlation Analysis

The correlation analysis indicated statistically significant positive linear relationships between AVCS values, evaluated based on the native phase and AVCS values estimated based on angiographic phase of MSCT examination: r AVCS_native3.0_ vs. AVCS_CTA0.6 500 HU_—0.85, r AVCS_native3.0_ vs. AVCS_CTA0.6 600 HU_—0.80, r AVCS_native2.0_ vs. AVCS_CTA0.6 500 HU_—0.85, r AVCS_native2.0_ vs. AVCS_CTA0.6 600 HU_—0.78.

### 3.6. Regression Analysis

Based on the univariate regression analysis, mathematical formulations were determined, allowing for AVCS native calculation based on known AVCS values, estimated based on MSCT angiographic phase:

AVCS_native3.0_ = 813.920 + 1.510 AVCS_CTA0.6 500 HU_.

AVCS_native3.0_ = 1235.863 + 1.817 AVCS_CTA0.6 600 HU_.

AVCS_native2.0_ = 797.471 + 1.393 AVCS_CTA0.6 500 HU_.

AVCS_native2.0_ = 1228.310 + 1.650 AVCS_CTA0.6 600 HU_.

The highest fit index in the case of the above equations, which was R2 = 0.710, characterized AVCS_native3.0_ value prediction based on AVCS_CTA0.6 500 HU_ estimated values. A slightly lower fit index in the case of the above equations, which was R2 = 0.708, characterized AVCS_native3.0_ value prediction based on AVCS_CTA0.6 600 HU_ estimated values.

Based on multifactorial regression analysis, the models were developed, allowing for the more precise prediction of actual AVCS, since a known estimated AVCS considers anthropometric parameters (age, gender, and BMI). Statistically significant models obtained in regression analysis are presented in Table 4.

The highest fit index in case of the analyzed models, indicating the best prediction, was found in the following models:

AVCS_native3.0_ = 1359.693 + 1.435 AVCS_CTA0.6 500 HU_ − 952.227 female (R2 = 0.737);

AVCS_native2.0_ = 1305.326 + 1.324 AVCS_CTA0.6 500 HU_ − 886.069 female (R2 = 0.736).

Both the above models indicate an independent, statistically significant decrease in actual AVCS value in the case of the estimation of its value in women in comparison with estimation in men.

### 3.7. Predictive Accuracy Analysis

Using sensitivity and specificity analysis, the accuracy of AVCS values was evaluated. These values were estimated based on the angiographic phase of MSCT examination as a predictive index of severe aortic stenosis probability, based on actual AVCS values (in accordance with AVCS_native3.0_). The complete results of sensitivity and specificity analysis of the AVCS criteria, determined based on ROC, are presented in Table 5.

In the group of women, the highest prediction accuracy was obtained when assuming AVCS_CTA0.6 500 HU_ ≥ 1569.00 value as the predictor of highly probable severe aortic stenosis. The accuracy of such an assumed criterion was 100%. However, in the group of men, the highest prediction accuracy was obtained when assuming the AVCS_CTA0.6 600 HU_ ≥ 1234.00 value as the predictor of highly probable severe aortic stenosis and AVCS_CTA0.6 600 HU_ ≥ 899.10 as the predictor of probable severe aortic stenosis. The accuracy of both these criteria was 92.3%. The remaining assumed criteria showed over 60% accuracy.

### 3.8. Potential Reduction of Ionizing Dose Analysis

The amount of a potential reduction in DLP ionizing dose in case of AVCS value estimation based on angiographic phase regarding possible omission of the native phase of examination was, on average, 30.50 ± 26.85 mGy. The above reduction amounts to 4.45 ± 1.54% of the total dose used in the MSCT examination of the heart and large vessels before TAVI. When limiting the study to the aortic valve only (examination involving the native phase dedicated to AVCS evaluation and angiographic phase dedicated to morphological evaluation of aortic ostia, excluding the angiographic phase, dedicated to the evaluation of all potential arterial accesses to TAVI procedure) the above dose reduction constitutes 11.03 ± 7.96% of the dose of this MSCT examination. No statistically significant differences were found regarding the size of a potential reduction in DLP ionizing radiation dose in the case of AVCS-estimated values based on the angiographic phase, due to the possible omission of a native phase between subgroups of the patients divided in accordance with their gender, body mass and age. The discussed dose reduction was statistically insignificantly higher in women than in men, in overweight and obese patients than in the patients with normal body weight and in senile patients rather than in the elderly. The potential reduction in ionizing radiation dose because of AVCS estimation in the study group and selected study subgroups is presented in Table 6.

## 4. Discussion

The performed examinations indicate that relying solely on the angiographic phase of MSCT examination of the heart and large vessels, it is possible to conclusively estimate the aortic valve calcium score. The increase in the calcification detection threshold from a standard level of 130 HU by the Agatston algorithm, used for actual AVCS calculation based on a native phase of MSCT examination to 500 HU or 600 HU value in angiographic phase of the MSCT examination, allows for the differentiation of some calcifications from the contrasted lumen of left ventricular outflow tract and/or aortic root. The estimated AVCS value is lower than the actual value due to the lost, in this method, calcifications of density between 130 and 500 or 600 HU. However, this study has proved that the obtained values of estimated AVCS strongly correlate with actual AVCS values (r between 0.78 and 0.85) and, using regression equations, recalculation of the estimated values into actual values can be performed with 70% fitting and, having considered the gender of the patients, with 73% fitting. Analogically, the applied method of AVCS estimation allows for evaluation of probability of severe aortic stenosis development and is typically performed based on AVCS actual value measured in a native phase of MSCT examination in axial reconstruction of 3.0 mm slice thickness. The performed ROC analysis has indicated cut-off points of estimated AVCS reflecting the classification criteria of the probability of severe aortic stenosis based on AVCS measured in the native phase. The performed sensitivity/specificity analysis determined the predicted accuracy of the resulting cut-off points of the estimated AVCS within the range of 62.5% and 100.0%.

Here, it appears necessary to comment on the increased calcification detection threshold in the angiographic phase of MSCT examination in the proposed method of AVCS estimation. A slightly higher compatibility between estimated and actual values of AVCS was obtained using the calcification detection threshold set at 500 HU. The increase in detection threshold from 500 HU to 600 HU, in turn, allowed for estimation to be performed in 98.04% of the examinations in comparison with the initial figure which was 76.47%. From a practical point of view, it seems that, while performing AVCS estimation, based on angiographic phase of MSCT examination, the lowest detection threshold, allowing for its performance, should be aimed for.

The applied AVCS estimation method may be of significant clinical importance. Thanks to its application, it has become possible to retrospectively analyze qualification examinations for the TAVI procedure that were performed before the popularization of the native phase, dedicated to AVCS evaluation in these types of examinations. Moreover, this method can be used to reliably estimate AVCS after any thoracic aorta CTA examination or even chest CT scan. Furthermore, as this study shows, the value estimation of AVCS based on the angiographic phase of MSCT examination, due to the omission of its native phase, is associated with a markedly lower ionizing radiation dose in MSCT examinations, corresponding with ALARA, the main radioprotection principle (As Low as Reasonably Achievable). The amount of a potential reduction in ionizing radiation dose expressed by DLP in the case of the discussed method of AVCS value estimation in the study group was, on average, 30.50 ± 26.85 mGy, which accounts for 4.45 ± 1.54% of the total dose in the MSCT examination of the heart and large vessels before TAVI and a 11.03 ± 7.96% dose concerning the heart region. The radiation dose reduction amount was independent of age, gender, and BMI.

The notion of AVCS evaluation based on angiographic phase of the examination has not been widely discussed in the currently available studies. Alqahtani et al., in their studies, assessed the possibility to estimate AVCS based on the MSCT examination of coronary arteries, assuming the calcification detection threshold based on ROI density measurement parameters placed at the ascending aorta, i.e., based on the mean and standard deviation of the abovementioned density. The calcification detection threshold was calculated based on a mathematical formula: mean + 2x standard deviation [17]. Similar to our study, it has been concluded that AVCS estimation, based on the angiographic phase of MSCT examination is possible and its results are reliable. The method used by Alqahtani et al. to estimate the AVCS is a method that requires repeatable and precise localization of ROI in the ascending aorta. This may result in greater variability of the obtained estimation results of this method in comparison to our method.". Bettinger et al., in their study, used AVCS estimated based on angiographic phase of MSCT examination, whose aim was to find the most precise prediction threshold for paravalvular leak after TAVI. In the cited above study AVCS was estimated using as many as 6 various thresholds of increased calcification predictions associated with aortic valve in the angiographic phase of MSCT examination: 650 Hu, 850 HU, 1.25 × LA, 1.5 × LA, LA + 50 and LA + 100. LA was the density in the lumen of aortic valve annulus in the angiographic phase of the examination. In these studies, it was indicated that the applied LA + 100 calcification detection threshold is characterized by the highest predictive value for paravalvular leak after TAVI [18]. However, in relatively older studies, Mühlenbruch et al. denied the feasibility of reliable AVCS estimation using the angiographic phase of MSCT examination. The calcium detection threshold in the angiographic phase of MSCT in this study was set at a markedly lower level, 350 HU [19].

However, the authors stress that analogous studies should also be mentioned, as they concern the attempts to estimate the coronary artery calcification score (CACS), solely based on the angiographic phase of MSCT examination. These types of studies were performed, among the others, by Mylonas et al., proving that CACS evaluation, based only on the MSCT angiography scan, is possible and correlates well with CACS evaluated routinely based on non-contrast enhanced examination. In their studies, the same calcification detection threshold was suggested, as in the presented studies by Alqahtani et al. [17,20].

Studies on various methodological aspects of AVCS evaluation appear crucial when considering the growing clinical importance of AVCS, especially in patients with aortic valve stenosis [12,21]. As mentioned before, based on the guidelines of the European Society of Cardiology, AVCS has been considered as the parameter that can be used to differentiate the degrees of aortic stenosis with the aortic ostium surface area below 1.0 cm^2^, a low gradient (<40 mmHg) and maintained left ventricular ejection fraction. AVCS_native3.0_ values over 2000 in men and 1200 in women [13] were criteria of highly probable severe aortic stenosis. Recently published multicenter studies by Pawade et el. confirmed the above criteria, obtaining, in the analysis of probable severe aortic stenosis, the thresholds of 2062 Agatston units in men and 1377 Agatston units in women [22]. Ren et al. proved that AVCS evaluation is a reliable marker in the evaluation of AS severity, also in patients with bicuspid aortic valve stenosis [21]. Scientific literature also discusses the significance of the AVCS onset value as a prognostic factor in patients who underwent the TAVI procedure [22,23].

However, the research carried out in recent years also stresses the role of AVCS evaluation in clinical conditions other than aortic stenosis. It has been suggested that, in patients without aortic valve stenosis, AVCS may be treated as the marker of atherosclerosis development process [24]. Higher values of AVCS are associated with higher risk of cardiovascular events in the future both in patients with cardiovascular diseases as well as in patients without clinical symptoms of cardiovascular disease [25]. Galąska et al., in their studies, showed that AVCS may even serve as a marker of genetic predisposition to hypercholesterolemia [26].

This study exemplifies the attempt at the optimization of the AVCS methodology evaluation using computed tomography. Another possible direction that scientific studies may follow concerning the AVCS methodology evaluation is its evaluation by other means of diagnostic imaging. Gillis et al. suggested an ultrasound evaluation of AVCS. The AVCS value, evaluated by echocardiography, correlated with the AVCS value, typically evaluated using non-contrast enhanced MSCT [27]. d’Humières T. et al. developed ultrasound AVCS evaluation equally well, correlating with the actual level of aortic valve calcification using less commonly applied three-dimensional echocardiography [28]. However, Le Ven et al. showed that the tissue characteristics of aortic ostium, including a quantitative participation of mineralized, fibrous, and lipid-rich elements may be successfully measured using a multiparametric imaging method of magnetic resonance with good-to-perfect precision, comparable with a histopathological examination [29].

Apart from the issues closely related to the aim of this study the obtained results also indirectly indicate the differences in AVCS between the genders. The performed regression analysis stresses the improvement of fit indices of the obtained mathematical formula, allowing for actual AVCS evaluation based on AVCS estimated values based on angiographic phase of MSCT examination when considering the gender of a studied patient. Female gender enforces correction of the calculated value by a few hundred units of HU, depending on the selected phase and estimation method. The obtained results appear to be in line with the documented fact of lower AVCS values in women. Simard et al. documented that, in patients with tricuspid aortic valve, when the severity of stenosis is similar, women, in comparison with men, exhibit a lower course of valve calcification advancement, but with a higher degree of its fibrosis [30]. Thaden et al. indicated, however, that, for the same degree of aortic stenosis severity, women show lower AVCS and lower mass of aortic valve in comparison with men, and this relationship is independent of valve morphological features. Moreover, AVCS correlates with aortic valve mass [31]. Lower AVCS in women was also observed in the general population. Koshkelashvili et al. documented higher AVCS in men than in women in the general population aged ≥ 65, but only in the case of Caucasian race [32]. Galas et al. showed higher values of AVCS in men in comparison with women in the group of patients aged ≥ 60 diagnosed due to thoracic pain using MSCT examination of coronary arteries [33].

The basic limitation of the methodology is the constant threshold of calcium density in the angiographic phase (500 or 600 HU). A variable density threshold “mean of blood pool density + standard deviation of blood pool density” would probably be better. However, different approaches are used in the literature. The choice of a specific, fixed threshold of calcium density in the angiographic phase was conditioned by the intention to test the simplest methodological approach. The calcium density threshold in the angiographic phase, based on the measurement of the mean and standard deviation of the blood pool density is a value whose measurement depends on many factors, including longitudinal measurement location (different value when measured in LA, LV, LVOT, aortic bulb, STJ point, ascending aorta or descending aorta), transverse location of measurement (median, eccentric, from the entire outline of the anatomical structure filled with blood), the size and shape of the ROI used for measurement, as well as dependent on the researcher making the measurement (intra-observer and inter-observer variability).

Another important limitation of the study is the small number of studies included in the project. Increasing the size of the study group would be valuable. However, it should be noted that the size of the group was considered in the algorithms of the performed statistical analyses.

Other limitations of the study include: performing the study only in Caucasian individuals; the single-center nature of the study limited the analysis to exams performed on one device in a given MSCT laboratory; the small size of the group of people with a bicuspid aortic valve (only 2 patients); failure to include in the analysis the MSCT exams performed with using lower kilo-voltages of the X-ray tube (100 kV, 80 kV); limited radiation dose characteristics in the analyzed studies using acquisition parameters provided by the CT instead of taking into account the size-specific dose estimation (SSDE); subjective selection of the MSCT phases, on the basis of which the AVCS was assessed. Moreover, the characteristics of the studied group lack information on the age of the menopause of the studied women, information on the medications used, and the results of the control echocardiographic examination. In the opinion of the authors, the above limitations do not significantly reduce the potential usefulness of the conducted research. The results should, therefore, be used as indicators for further research.

## 5. Conclusions

1. Relying solely on the angiographic phase of MSCT examination of the heart and large vessels, it is possible to conclusively estimate the aortic valve calcium score.
2. The AVCS estimation based on the angiographic phase of the MSCT study, due to the omission of the native phase of the study, results in a lower dose of ionizing radiation.

## Figures and Tables

**Table 1:** Clinical characteristics of the study group of patients (*n* = 51).

	**X**	**Me**	**Min**	**Max**	**SD**
age [years]	78.59	81.00	62.00	89.00	5.72
BMI [kg/m^2^]	27.68	28.26	22.20	33.33	3.03
total cholesterol [mg/dL]	210.36	206.00	117.00	445.00	57.43
LDL cholesterol [mg/dL]	130.84	125.00	46.00	218.00	47.00
HDL cholesterol [mg/dL]	47.52	47.00	33.00	69.00	9.04
triglicerides [mg/dL]	218.28	178.50	66.00	875.00	104.23
glucose [mg/dL]	130.31	114.00	75.00	312.00	50.83
systolic BP [mmHg]	137.50	135.00	105.00	166.00	19.38
diastolic BP [mmHg]	84.85	84.00	62.00	115.00	15.25
creatinine [mg/dL]	1.24	1.23	0.82	1.54	0.23
eGFR [mL/(min × 1.73 m^2^)]	63.59	63.50	45.00	101.00	9.95
	***n***		**%**		
age					
elderly	9		17.65		
senile	42		82.35		
gender					
male	26		50.98		
female	25		49.02		
body mass					
normal	11		21.57		
overweight	31		60.78		
obesity	9		17.65		
CVD					
diabetes	15		29.41		
dyslipidemia	28		54.90		
arterial hypertension	46		90.20		

BMI—body mass index; BP—blood pressure; CVD—cardiovascular diseases; eGFR—estimated glomerular filtration rate; HDL—high-density lipoprotein; LDL—low-density lipoprotein; Max—maximum value; Min—minimum value; *n*—number of patients; SD—standard deviation; X—arithmetic average.

**Table 2:** Standard parameters of aortic valve evaluation in a MSCT procedure qualifying for TAVI in the study group (*n* = 51).

	***n***		**%**		
number of aortic valve cusps					
2	2		3.92		
3	49		96.08		
	**X**	**Me**	**Min**	**Max**	**SD**
aortic valve annulus					
maximum measurement [mm]	26.64	26.00	23.00	37.00	3.21
minimum measurement [mm]	20.36	20.00	17.00	27.00	2.33
mean measurement [mm]	23.50	22.50	20.50	32.00	2.62
aortic root					
maximum measurement [mm]	33.52	33.00	28.00	43.00	4.58
minimum measurement [mm]	30.80	30.00	26.00	40.00	4.13
mean measurement [mm]	32.16	31.50	27.50	41.50	4.28
height [mm]	19.88	18.00	14.00	28.00	3.79
distance from coronary ostia to aortic valve annulus					
left coronary artery [mm]	13.16	13.00	10.00	18.00	2.08
right coronary artery [mm]	14.08	14.00	12.00	18.00	1.89

Max—maximum value; Me—median; Min—minimum value; *n*—number of patients; SD—standard deviation; X—arithmetic average.

**Table 3:** AVCS and the parameters characterizing ionizing radiation dose in a MSCT procedure qualifying for TAVI in the study group (*n* = 51).

	**X**	**Me**	**Min**	**Max**	**SD**
native aortic valve calcium score (AVCS_native_)					
3.0 mm slice thickness evaluation	3690.54	3022.40	1052.90	9453.40	2378.82
2.0 mm slice thickness evaluation	3457.03	2858.10	1035.40	9148.80	2190.81
	*n*		%		
probability of severe aortic stenosis (estimated in accordance with AVCS_native3.0_)					
highly probable (M ≥ 3000, F ≥ 1600)	30		58.82		
probable (M ≥ 2000, F ≥ 1200)	45		88.23		
improbable (M ≥ 1600, F ≥ 800)	3		5.88		
	**X**	**Me**	**Min**	**Max**	**SD**
estimated on the basis of angiographic phase aortic valve calcium score (AVCS_CTA0.6_)					
500 HU calcification detection threshold	2068.62	1608.00	276.50	5620.00	1422.23
600 HU calcification detection threshold	1372.39	1239.40	119.70	3634.50	1044.53
radiation dose in a native phase dedicated to AVCS evaluation					
CTDIvol [mGy]	2.35	1.87	0.13	13.17	2.55
DLP [mGycm]	30.50	23.30	11.00	143.60	26.85
radiation dose in angiographic phase dedicated to morphological evaluation of aortic ostium					
CTDIvol [mGy]	29.58	16.29	4.72	245.00	45.63
DLP [mGycm]	324.18	220.00	46.30	2058.00	328.41
total radiation dose of MSCT examination of heart and large vessels					
DLP [mGycm]	697.94	554.00	190.00	2380.00	472.17

Max—maximum value; Me—median; Min—minimum value; *n*—number of patients; SD—standard deviation; X—arithmetic average.

**Table 4:** Mathematical formulae allowing for the calculation of a native AVCS based on AVCS values estimated based on angiographic phase of a MSCT procedure qualifying for TAVI in the study group (*n* = 51) obtained in a regression analysis. (**A**) AVCSnative evaluated at 3.0 slice thickness. (**B**) AVCSnative evaluated at 2.0 slice thickness.

**A.** AVCS_native_ evaluated at 3.0 slice thickness.		
**Parameters Considered in the Model**	**Mathematical Equation**	**Parameters of Model Fitting**
dependent variable: AVCS_native3.0_ independent variables: AVCS_CTA0.6 500 HU_, intercept	AVCS_native3.0_ = 813.920 + 1.510 AVCS_CTA0.6 500 HU_	model *p* < 0.000 *p* AVCS_CTA0.6 500 HU_: 0.000 intercept *p* 0.043 model R2: 0.710
dependent variable: AVCS_native3.0_ independent variables: AVCS_CTA0.6 600 HU_, intercept	AVCS_native3.0_ = 1.235.863 + 1.817 AVCS_CTA0.6 600 HU_	model *p* < 0.000 *p* AVCS_CTA0.6 500 HU_: 0.000 intercept *p* 0.001 model R2: 0.625
dependent variable: AVCS_native3.0_ independent variables: AVCS_CTA0.6 500 HU_, gender, intercept	AVCS_native3.0_ = 1359.693 + 1.435 AVCS_CTA0.6 500 HU_ − 952.227 female	model *p* < 0.000 *p* AVCS_CTA0.6 500 HU_: 0.000 gender *p*: 0.035 intercept *p* 0.043 model R2: 0.737
dependent variable: AVCS_native3.0_ independent variables: AVCS_CTA0.6 600 HU_, gender, intercept	AVCS_native3.0_ = 1527.117 + 1.750 AVCS_CTA0.6 600 HU_ − 415.270 female	model *p* < 0.000 *p* AVCS_CTA0.6 500 HU_: 0.000 gender *p* 0.034 intercept *p* 0.001 model R2: 0.624
dependent variable: AVCS_native3.0_ independent variables: AVCS_CTA0.6 500 HU_, gender, BMI, intercept	AVCS_native3.0_ = 2333.771 + 1.474 AVCS_CTA0.6 500 HU_ − 974.063 female − 37.563 BMI	model *p* < 0.000 *p* AVCS_CTA0.6 500 HU_: 0.000 gender *p* 0.033BMI *p* 0.635 intercept *p* 0.270 model R2: 0.731
dependent variable: AVCS_native3.0_ independent variables: AVCS_CTA0.6 600 HU_, gender, BMI, intercept	AVCS_native3.0_ = 3386.066 + 1.876 AVCS_CTA0.6 600 HU_ − 428.299 female 73.450 BMI	model *p* < 0.000 *p* AVCS_CTA0.6 500 HU_: 0.000 gender *p* 0.033 BMI *p* 0.416 intercept *p* 0.149 model R2: 0.621
**B.** AVCS_native_ evaluated at 2.0 slice thickness.		
**Parameters Considered in the Model**	**Mathematical Equation**	**Parameters of Model Fitting**
dependent variable: AVCS_native2.0_ independent variables: AVCS_CTA0.6 500 HU_, intercept	AVCS_native2.0_ = 797.471 + 1.393 AVCS_CTA0.6 500 HU_	model *p* < 0.000 *p* AVCS_CTA0.6 500 HU_: 0.000 intercept *p* 0.033 model R2: 0.708
dependent variable: AVCS_native2.0_ independent variables: AVCS_CTA0.6 600 HU_, intercept	AVCS_native2.0_ = 1.228.310 + 1.650 AVCS_CTA0.6 600 HU_	model *p* < 0.000 *p* AVCS_CTA0.6 500 HU_: 0.000 intercept *p* 0.000 model R2: 0.607
dependent variable: AVCS_native2.0_ independent variables: AVCS_CTA0.6 500 HU_, gender, intercept	AVCS_native2.0_ = 1305.326 + 1.324 AVCS_CTA0.6 500 HU_ − 886.069 female	model *p* < 0.000 *p* AVCS_CTA0.6 500 HU_: 0.000 gender *p* 0.034 intercept *p* 0.003 model R2: 0.736
dependent variable: AVCS_native2.0_ independent variables: AVCS_CTA0.6 600 HU_, gender, intercept	AVCS_native2.0_ = 1484.181 + 1.591 AVCS_CTA0.6 600 HU_ − 364.820 female	model *p* < 0.000 *p* AVCS_CTA0.6 500 HU_: 0.000 gender *p* 0.038 intercept *p* 0.001 model R2: 0.605
dependent variable: AVCS_native2.0_ independent variables: AVCS_CTA0.6 500 HU_, gender, BMI, intercept	AVCS_native2.0_ = 2206.638 + 1.360 AVCS_CTA0.6 500 HU_ − 906.274 female − 34.757 BMI	model *p* < 0.000 *p* AVCS_CTA0.6 500 HU_: 0.000 gender *p* 0.033 BMI *p* 0.635 intercept *p* 0.260 model R2: 0.730
dependent variable: AVCS_native2.0_ independent variables: AVCS_CTA0.6 600 HU_, gender, BMI, intercept	AVCS_native2.0_ = 3323.035 + 1.716 AVCS_CTA0.6 600 HU_ − 377.709 female − 72.656 BMI	model *p*: <0.000 *p* AVCS_CTA0.6 500 HU_: 0.000 gender *p*: 0.037 BMI *p*: 0.393 intercept *p* 0.134 model R2: 0.603

**Table 5:** Prediction of severe aortic stenosis, based on AVCS values, estimated based on angiographic phase of a MSCT procedure qualifying for TAVI in the study group (*n* = 51).

Probability of Severe Aortic Stenosis (in Accordance with AVCS_native3.0_)	Cut-off Point of Estimated AVCS based on ROC Analysis	Test Evaluation Parameters		
		Sensitivity	Specificity	Accuracy
highly probable (M ≥ 3000)	AVCS_CTA0.6 500 HU_ ≥ 1577.20.	1.000	0.882	0.913
	AVCS_CTA0.6 600 HU_ ≥ 1234.00.	1.000	0.889	0.923
highly probable (K ≥ 1600)	AVCS_CTA0.6 500 HU_ ≥ 1569.00.	1.000	1.000	1.000
	AVCS_CTA0.6 600 HU_ ≥ 746.40.	1.000	0.700	0.813
probable (M ≥ 2000)	AVCS_CTA0.6 500 HU_ ≥ 1183.50.	0.500	1.000	0.913
	AVCS_CTA0.6 600 HU_ ≥ 899.10.	0.500	1.000	0.923
probable (K ≥ 1200)	AVCS_CTA0.6 500 HU_ ≥ 746.40.	0.167	0.900	0.625
	AVCS_CTA0.6 600 HU_ ≥ 746.40.	0.308	1.000	0.625
improbable (M < 1600)	AVCS_CTA0.6 500 HU_ < 706.00	0.950	0.333	0.870
	AVCS_CTA0.6 600 HU_ < 502.00	0.957	0.333	0.885
improbable (K < 800)	uncertain evaluation due to lack of women with AVCS < 800 in the study group			

**Table 6:** Potential reduction in ionizing radiation dose because of AVCS estimation, based on the angiographic phase of an MSCT procedures qualifying for TAVI in the study group (*n* = 51).

	Radiation Dose—DLP [mGycm]			Potential Reduction of Radiation Dose [%]	
group	native phase dedicated to AVCS evaluation	angiographic phase dedicated to morphological evaluation of aortic ostium	total MSCT examination of heart and large vessels	regarding examination limited to angiographic phase dedicated to morphological evaluation of aortic ostium	regarding total MSCT examination of heart and large vessels
total study group	30.50 ± 26.85	324.18 ± 328.41	697.94 ± 472.17	11.03 ± 7.96	4.45 ± 1.54
male	36.10 ± 34.43	411.45 ± 417.16	846.42 ± 574.69	10.46 ± 9.94	4.04 ± 1.21
female	24.68 ± 14.06	233.43 ± 163.08	543.52 ± 267.80	11.64 ± 5.33	4.88 ± 1.75
normal body weight	31.59 ± 12.50	497.04 ± 286.49	915.27 ± 394.26	7.01 ± 2.97	3.70 ± 1.22
overweight	34.23 ± 32.65	320.07 ± 358.19	717.10 ± 509.76	11.53 ± 9.03	4.51 ± 1.47
obesity	16.32 ± 5.68	127.08 ± 74.24	366.33 ± 185.92	14.28 ± 6.63	5.16 ± 1.88
elderly age	34.14 ± 22.15	569.00 ± 619.81	758.11 ± 388.54	8.29 ± 5.01	4.39 ± 1.75
senile age	29.72 ± 27.93	271.72 ± 201.48	685.05 ± 491.39	11.63 ± 8.39	4.46 ± 1.52

## Data Availability

Study data can be made available upon documented request.
